# Supplementary material for: Causal Relationship between Aging and Anorexia Nervosa: A White-Matter-Microstructure-Mediated Mendelian Randomization Analysis
Source: Biomedicines. 2024 Aug 16;12(8):1874. doi: 10.3390/biomedicines12081874 (PMC11351342; doi:10.3390/biomedicines12081874)
Supplement: Supplementary file 1 [file biomedicines-12-01874-s001.zip › Supplementary Figure/Figure S4/Singe SNP plot of MR analysis for TL to ubm-b-1709.pdf]

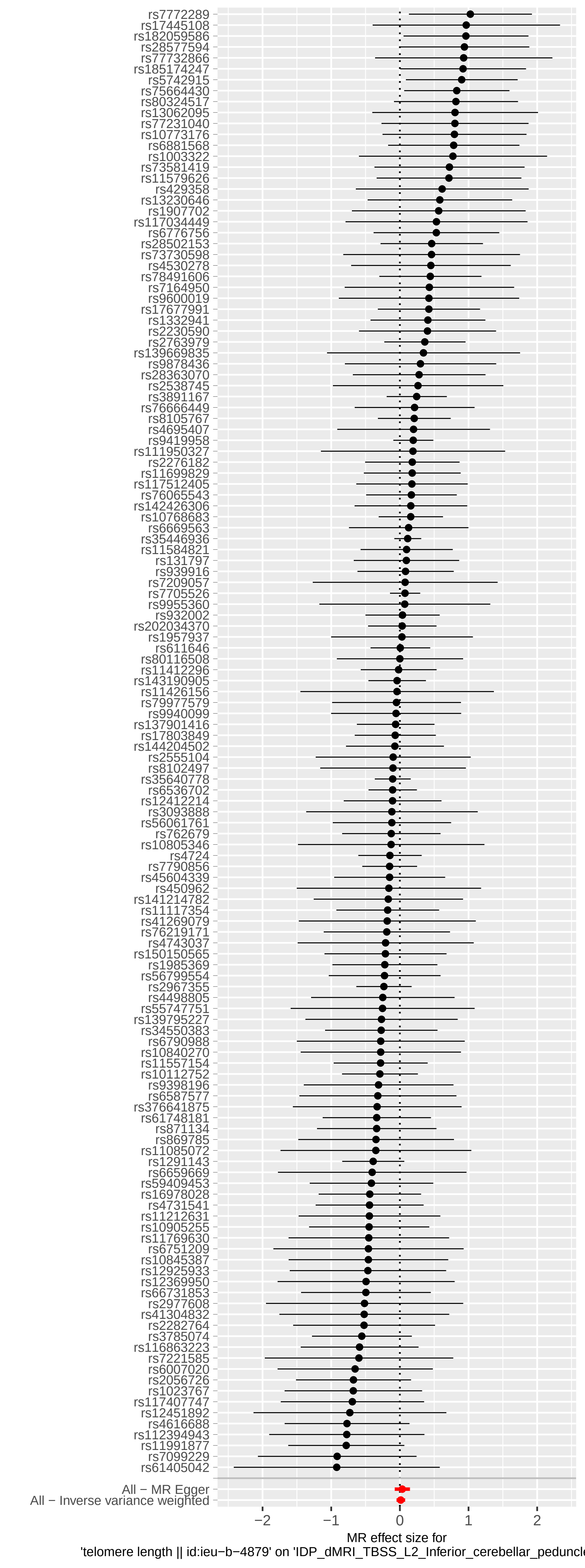
rs7772289
rs17445108
rs182059586
rs28577594
rs77732866
rs185174247
rs5742915
rs75664430
rs80324517
rs13062095
rs77231040
rs10773176
rs6881568
rs1003322
rs73581419
rs11579626
rs429358
rs13230646
rs1907702
rs117034449
rs6776756
rs28502153
rs73730598
rs4530278
rs78491606
rs7164950
rs9600019
rs17677991
rs1332941
rs2230590
rs2763979
rs139669835
rs9878436
rs28363070
rs2538745
rs3891167
rs76666449
rs8105767
rs4695407
rs9419958
rs111950327
rs2276182
rs11699829
rs117512405
rs76065543
rs142426306
rs10768683
rs6669563
rs35446936
rs11584821
rs131797
rs939916
rs7209057
rs7705526
rs9955360
rs932002
rs202034370
rs1957937
rs611646
rs80116508
rs11412296
rs143190905
rs11426156
rs79977579
rs9940099
rs137901416
rs17803849
rs144204502
rs2555104
rs8102497
rs35640778
rs6536702
rs12412214
rs3093888
rs56061761
rs762679
rs10805346
rs4724
rs7790856
rs45604339
rs450962
rs141214782
rs11117354
rs41269079
rs76219171
rs4743037
rs150150565
rs1985369
rs56799554
rs2967355
rs4498805
rs55747751
rs139795227
rs34550383
rs6790988
rs10840270
rs11557154
rs10112752
rs9398196
rs6587577
rs376641875
rs61748181
rs871134
rs869785
rs11085072
rs1291143
rs6659669
rs59409453
rs16978028
rs4731541
rs11212631
rs10905255
rs11769630
rs6751209
rs10845387
rs12925933
rs12369950
rs66731853
rs2977608
rs41304832
rs2282764
rs3785074
rs116863223
rs7221585
rs6007020
rs2056726
rs1023767
rs117407747
rs12451892
rs4616688
rs112394943
rs11991877
rs7099229
rs61405042
All − MR Egger
All − Inverse variance weighted
−2
−1
0
1
2
MR effect size for
'telomere length || id:ieu−b−4879' on 'IDP_dMRI_TBSS_L2_Inferior_cerebellar_peduncl
